# Quantitative Analysis of Charge Distribution in Bi-Emissive layer White Organic Light-Emitting Diodes with Two Fluorescent Dopants

**DOI:** 10.1038/s41598-018-21531-6

**Published:** 2018-02-16

**Authors:** Ji Young Kim, Woo Young Kim, Kok Wai Cheah

**Affiliations:** 1 Department of Physics, Hong Kong Baptist University, Kowloon Tong, Hong Kong; 2 Department of Electronic display engineering, Hoseo University, Asan, 31499 South Korea; 3 Department of Engineering Physics, McMaster University, Hamilton, Ontario L8S 4L7 Canada

## Abstract

This work seeks to establish a quantitative method which can estimate the holes and electrons ratio in the emission zones. We fabricated multilayered white organic light-emitting diodes (WOLEDs) with the device structure of ITO/NPB(80 nm)/MADN:BUBD-1(7%)(20 nm)/MADN:DCJTB(0.3%)(20 nm)/TPBi(X nm)/LiF(2 nm)/Al as a case study on the charge recombination distribution in the emissive layer. The result shows a trend in the charge recombination ratio depending on the electron transport layer thickness. We obtained an empirical relationship between electron transport layer thicknesses and emission ratio in EML. In addition, the electroluminescent spectra were analyzed by fitting a Gaussian distribution for the two emissive layers to calculate the intensity ratio of the energy transitions. The arrival time of hole and electrons from each electrode was determined using the thickness and mobility of TPBi as electron transport layer. From these initial results, we derived an empirical mechanism to meet with a linear relationship that can allow us to design custom- made WOLEDs.

## Introduction

There are many works in developing white organic light-emitting diodes (WOLEDs) technology for solid-state lighting applications. Most of the researches used to fabricate single-emitting layer (EML) or multi-EML WOLEDs. The methods can be separated into two types of optical color mixture. One is three primary colors of red, green and blue (RGB) combinations were simultaneously doped into the same host^1^, and the other is the two primary colors of blue, then either red, orange or yellow dopants were doped into each different host^2–4^. For single-EML, it has better color stability, easy fabrication; meanwhile the electroluminescence (EL) efficiency is relatively low^5,6^. Multi-EML WOLEDs have the merit of higher EL efficiency than that of single-EML WOLEDs, however its Commission Internationale de L’Eclairage (CIEx,y) coordinates are generally dependent upon driving voltage due to the shift of the exciton recombination zone^7,8^.

In this paper, a multi-EML WOLED with the structure of blue fluorescent EML/red fluorescent EML was presented. Fluorescent dopant systems have fabricated their capability to deliver pure white color coordinates as well as long life time^9,10^. However, the power efficiencies of these devices are limited. The use of fluorescent dopants limits the internal quantum efficiency to a maximum value of 25% due to well-known spin symmetry^11^. This work pursues to quantify the estimation of the emission ratio between blue and red EML by varying the thickness of transporting layers. As we know, emission ratio is extremely sensitive to many circumstances, we fabricated an uncomplicated multi-layered WOLED with the device structure which of ITO/NPB(80 nm)/MADN:BUBD-1(7%)(20 nm)/MADN:DCJTB(0.3%)(20 nm)/TPBi(X nm)/LiF(2 nm)/Al. The EL spectra of devices were fitted with a Gaussian distribution and obtained a relationship between thickness of HTL/ETL and ratio of two emission peaks. In addition, we have theoretically calculated the arrival time of holes and electrons in the emissive layer by considering the carrier mobility and thickness of each organic layers. Therefore, we show how to theoretically quantify the emissive charge distribution ratio in WOLEDs with multiple emissive layers by varying the thickness of hole and electron transport layers. An empirical equation can be derived from this method to estimate the emissive ratio of the recombination zone that can allow us to design custom-made WOLEDs. However, based on this research, this empirical equation can only be demonstrated with identical structures with same materials as this paper due to differences of charge carrier mobility and inappropriate energy barriers. There are a few previous studies which related on this topic utilizing several different methods^12,13^.

## Methods

**Figure 1:**
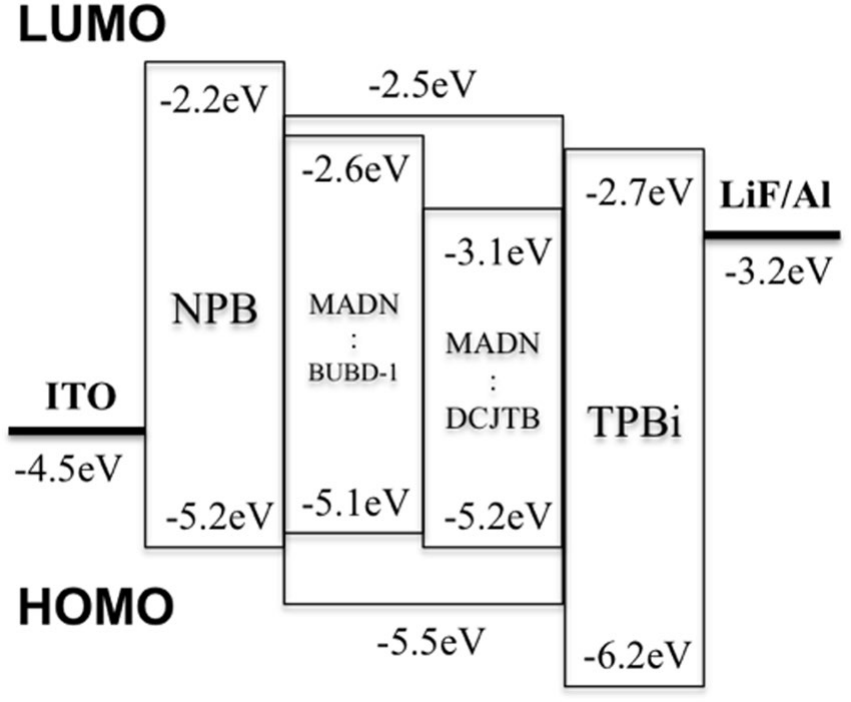
The Configuration and the energy diagram of the devices.

The device structure and energy diagram of the fabricated WOLED are shown in Fig. 1. The lowest unoccupied molecular orbital (LUMO) and highest occupied molecular orbital (HOMO) values of the organic materials NPB (−2.2 eV/−5.2 eV)^14^, BUBD-1 (−2.6 eV/−5.1 eV), MADN (−2.5 eV/−5.5 eV)^15^, DCJTB (−3.1 eV/−5.2 eV)^16^ and TPBi (−2.7 eV/−6.2 eV)^17^ are obtained from this paper. All the devices appeared here have the configuration of ITO/NPB/MADN:BUBD-1/MADN:DCJTB/TPBi/LiF/Al, in which NPB is (N,N′- bis (naphthalen-1-yl)-N,N′-bis (phenyl)-benzidine) as a hole transport layer, MADN is (2-methyl-9,10-bis (naphthalen-2-yl) anthracene) used as a host, BUBD-1 and DCJTB (4-(dicyanomethylene)-2-*t*-butyl-6(1,1,7,7-tetramethyljulolidyl-9-enyl)-4H-pyran) are employed as a blue and red fluorescent dopant, respectively. TPBi serves (2,2′,2′′-(1,3,5-benzinetriyl)-tris (1-phenyl-1-Hbenzimidazole)) as an electron transport layer. TPBi has a low HOMO energy level (−6.2 eV) that used as a hole-blocking material^18^. The emission of TPBi was completely inhibited and could not be detected even with increased the driving voltage. Therefore, it is a suitable electron transporting material for this work^19^. All energy level values obtained from the references except for BUBD-1. The energy level of BUBD-1 was in-house measured.

The devices were fabricated on glass substrates pre-coated with indium tin oxide (ITO) as the anode. Lithium fluoride (LiF) was deposited prior to forming aluminum (Al) cathode for better electron injection. After cleaning the substrates with a routine procedure^20^, the thin film deposition was carried out in a thermal evaporation chamber and the base pressure was 4.0 × 10^−6^ Torr. The active area of the devices is 10 *mm*^2^. The electroluminescent (EL) spectra and the CIE color coordinates were measured by PR650 spectrophotometer and the characteristics of current density–voltage-luminance (J-V-L) measurements were recorded using a Keithley 236 source meter.

## Results

### Multi-peak fitting with Gaussian function

**Figure 2:**
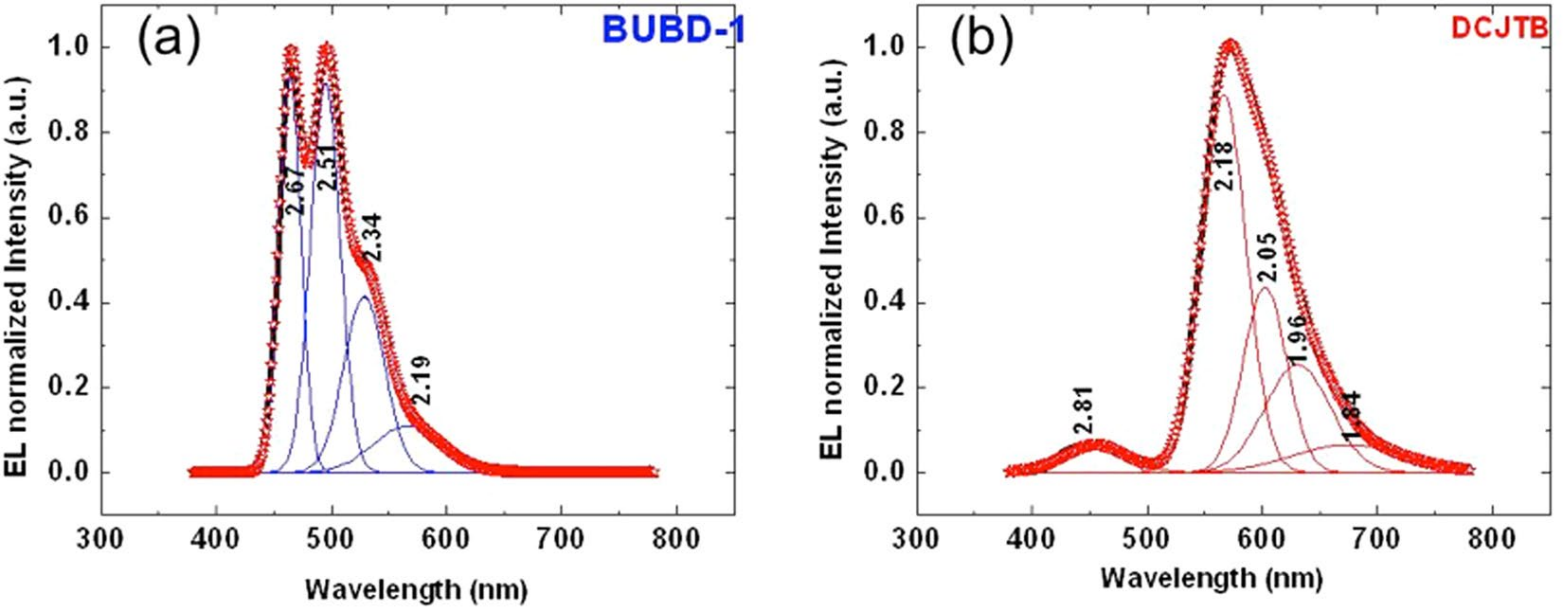
Multi-peak fitting with Gaussian function for EL spectrum of (**a**) MADN:BUBD-1(7%) and (**b**) MADN:DCJTB (0.3%). The EL spectra peaks are represented in electron volts.

There are a total of four emission peaks in MADN:BUBD-1(7%) EL spectrum: 464, 494, 529, and 567 nm of wavelength, corresponding to energy of 2.67, 2.51, 2.34, and 2.19 eV respectively. For MADN:DCJTB (0.3%) EL spectrum there are also 5 emission peaks: 451, 568, 604, 632, and 673 nm of wavelength which their energy of 2.75, 2.18, 2.05, 1.96, and 1.84 eV respectively as shown in Fig. 2. These peaks reflects the energy transitions of MADN:BUBD-1 and MADN:DCJTB in recombination zone. In order to obtain the emission ratio the EL spectrum was fitted with the peak functions of Gaussian distribution method and the EL spectrum was analyzed in the electron volt using the peak fitting functions as described in Fig. 2. For MADN:DCJTB(0.3%) peaks, it has a slight blue peak due to the low concentration of DCJTB dopant. Each energy transition was originated from the HOMO level which is the excited state relaxing through the relatively sharp and symmetrical peak centered wavelength, *λ* = *c*/*ν* = *ħc*/(Δ*E*), where c is the speed of light, *ν* is the photon frequency on the ground state and *ħ* is the Plank constant^21^.

Varying the thickness of hole and electron transport layers to change the ratio of BUBD-1 (blue) and DCJTB (red) emission peaks in the recombination zone. We utilized a multi-peaks Gaussian fitting method to find the emission ratio.

In this work, the structure of WOLED is ITO/NPB(80 nm)/MADN:BUBD-1(7%)(20 nm)/MADN:DCJTB(0.3%) (20 nm)/TPBi(Xnm)/LiF(2 nm)/Al and the blue, red dopant concentration are optimized with 7% and 0.3%, respectively. All devices had fixed EML thicknesses consist of MADN:BUBD-1 (20 nm) and DCJTB(20 nm) due to higher efficiency and stability compared with other thicknesses. Table 1 summarizes the values of x, which is the thickness of ETL (TPBi).

**Table 1:** WOLED structures of devices with different thickness of TPBi in ETL (All devices were deposited with LiF = 2 nm, Al = 80 nm).

	HTL	EML1	EML2	ETL
Device A	NPB	MADN:BUBD-1 (7%)	MADN:DCJTB (0.3%)	TPBi
	(80 nm)	(20 nm)	(20 nm)	(5 nm)
Device B	NPB	MADN:BUBD-1 (7%)	MADN:DCJTB (0.3%)	TPBi
	(80 nm)	(20 nm)	(20 nm)	(20 nm)
Device C	NPB	MADN:BUBD-1 (7%)	MADN:DCJTB (0.3%)	TPBi
	(80 nm)	(20 nm)	(20 nm)	(35 nm)
Device D	NPB	MADN:BUBD-1 (7%)	MADN:DCJTB (0.3%)	TPBi
	(80 nm)	(20 nm)	(20 nm)	(50 nm)

**Figure 3:**
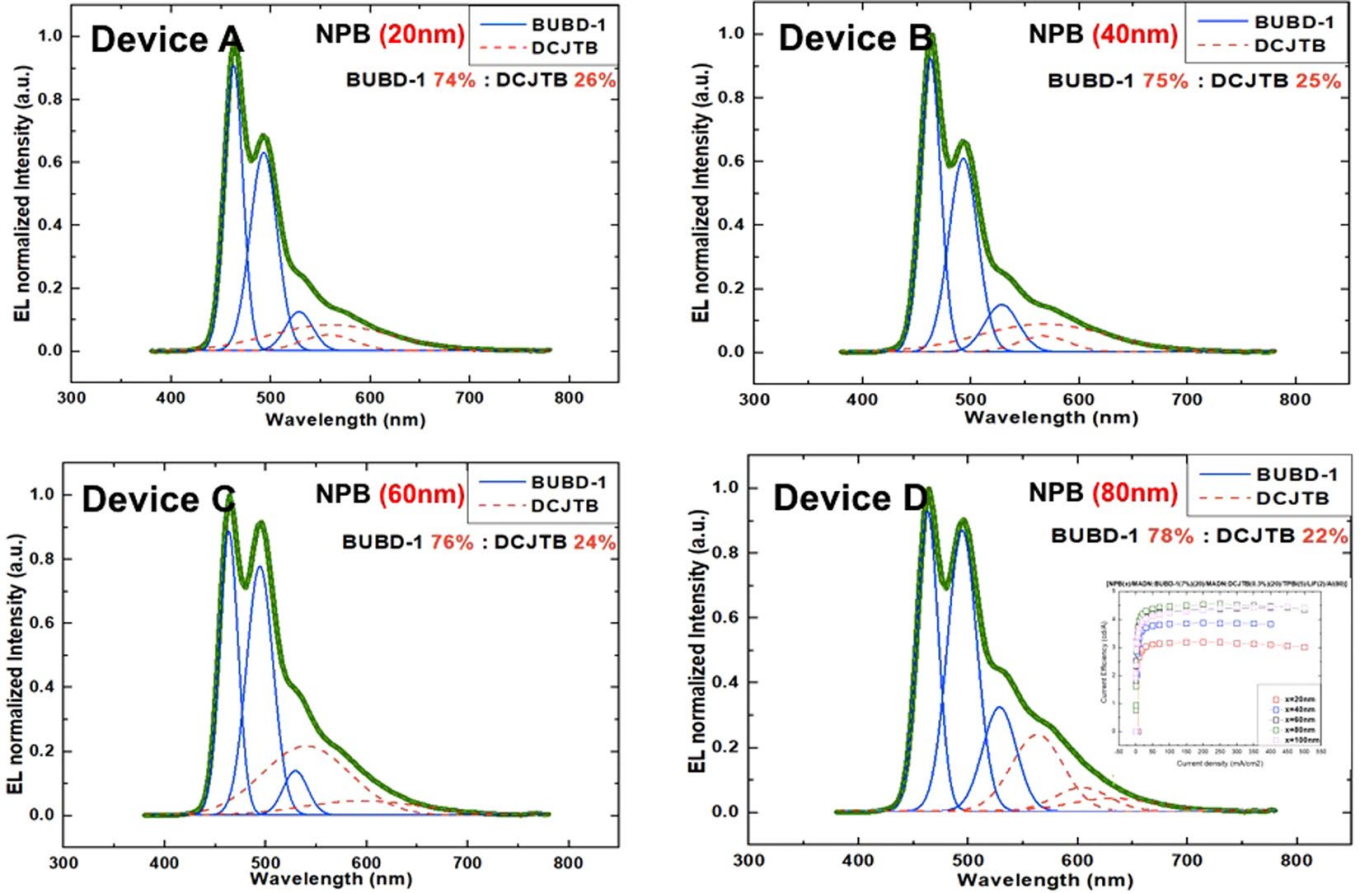
Normalized EL Intensity fitted with the Peak Functions of Gaussian Distribution of Device (**A**,**B**,**C** and **D**) at 5 V. The structure of these devices is ITO/NPB(x nm)/MADN:BUBD-1(7%)(20 nm)/MADN:DCJTB(0.3%)(20 nm)/TPBi (5 nm)/LiF(2 nm)/Al and current efficiency of Device (**A**) to (**D**).

Figure 3 shows the normalized EL spectra of device A to D at 5 V and there were five EL peaks at 415 nm, 464 nm, 494 nm, 529 nm, and 567 nm accorded with BUBD-1 which emitted blue color whereas the other emission peaks at 568 nm, 604 nm, 632 nm and 673 nm are corresponded to the MADN:DCJTB emissions.

The device A to D had different thickness HTL but EML and ETL thicknesses were fixed as 40 nm and 5 nm, respectively. These EL spectra explained the reason why we used 80 nm of ETL on this research. The ETL thickness was fixed as 5 nm and the HTL thicknesses were changed from 20 nm to 80 nm which gave stable blue and red emission with average ratio of 75% and 25% approximately. The emission ratio was derived from integrating the Gaussian distribution under each fitted peak area of the EL spectra in these OLED devices.

In Fig. 3 the relative emission intensities were included in the fitted area and can be estimated as the percentage of the total emission. The percentage of the average total emission 75:25 of Device A to D was explained by the slightly higher hole mobility of NPB than the electron mobility of TPBi. Therefore, we can realize that the HTL thickness did not influence on the emission ratio dramatically. In addition, Fig. 3 Device D shows 80 nm of HTL(NPB) thickness has better current efficiency and stability than any other devices.

**Figure 4:**
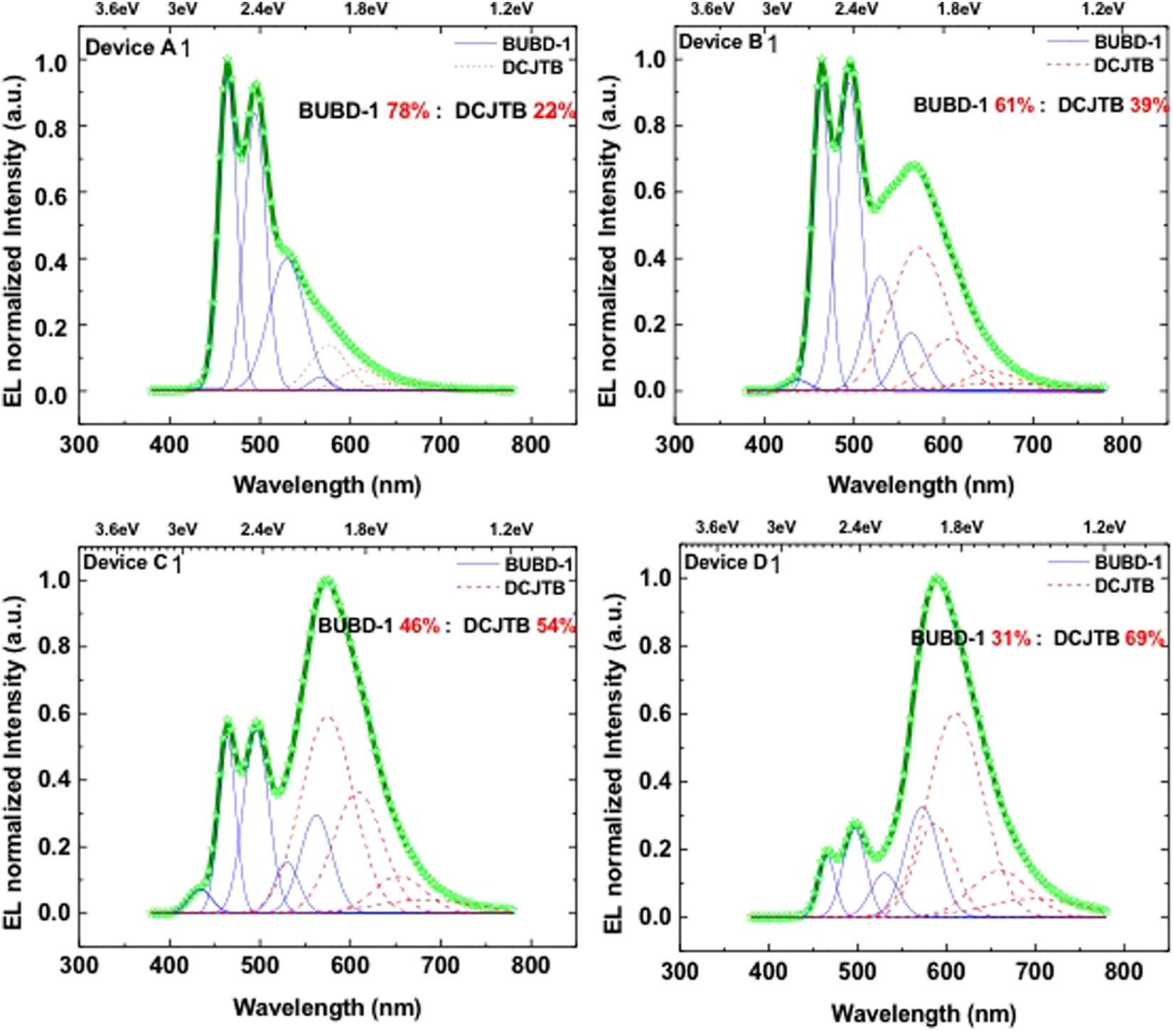
Normalized EL Intensity fitted with the Peak Functions of Gaussian Distribution of Device (**A1**,**B1**,**C1** and **D1**) at 5 V. The structure of these devices is ITO/NPB(80 nm)/MADN:BUBD-1(7%)(20 nm)/MADN:DCJTB(0.3%)(20 nm)/TPBi (x nm).

Figure 4 shows the Devices A1 to D1 had different ETL thicknesses but HTL and EML thicknesses were fixed as 80 nm and 40 nm, respectively.

Based on the EL spectra of Fig. 3, Device D has 78:22 of emission ratio with NPB 80 nm and TPBi 5 nm and we varied the ETL thickness from 5 nm to 50 nm so that the emission ratio is changed from 78:22 to 31:69 of emission ratio between BUBD-1(Blue):DCJTB(Red) under fixed HTL thickness, respectively. Figure 4 describes the peak fitting with Gaussian distribution as mentioned above. From the results, we can obtain the correlation between the thickness of transport layers and the ratio of color emission intensity.

### Correlation between the thickness of transport layers and the white color emission ratio

From the results above, we further fabricated other devices based on this fitting method and the relationship. The increase in TPBi thickness leads to increase in red emission from DCJTB. Therefore, the emission ratio between BUBD-1 and DCJTB is mostly influenced by thickness of TPBi using as ETL. The emission ratio was obtained by integrating Gaussian fittings for each peak and can be estimated as the percentage out of the total emission as shown in Fig. 4. Here, we can investigate the correlation between the thickness of transport layers and the white color emission ratio. As the thickness of the electron transport layer increased, the emission spectrum was shown to move from cool white light to more warm white color. From these results, we further fabricated other devices based on this fitting method and the relationship. It shows the correlation with thickness of HTL(NPB)/ETL(TPBi) and BUBD-1 emission ratio. The ratio of blue and red emission could vary from maximum 78% to minimum 29% in white color when TPBi thickness was changed from 5 nm to 50 nm due to the different thickness of layer and this can be influenced on optical micro-cavity that produces interference phenomena in the emission ratio^22^. This can be explained by the higher hole mobility of NPB compared to electron mobility of TPBi, the recombination zone was significantly affected by the thickness of TPBi.

This result provides experimental support the established of empirical relationship between the thickness of transport layer and the emission ratio. According to this theory, it is possible to estimate an emission zone along the emission ratio in EML using the mobility of each layers’ material and their thicknesses.

### Time of arrival

The charge carrier mobility depends on the electric field and thickness of each layer will allow us to calculate the ‘time of arrival’ of the carriers at each point. Assuming that the energy levels between layers remain unchanged across all organic layers at room temperature. Thus, the electric field (*E*) across the organic layers can be readily obtained from the bias voltage (*v*) and total thickness of organic layers (*d*_*org*_):

1 $$E=\frac{v}{{d}_{org}}$$

To verify the carrier drift velocity (*ν*_*d*_) is using the electric field as shown in Eq. (1) and the charge carrier mobility (*μ*) when the electric field is *E* ≥ 0.5 *MV*/*cm*

2 $${\nu }_{d}=\mu \cdot E$$

The charge carrier mobility (*μ*) is affected by the energetic disorder due to random orientation of dipoles in the organic layers^23^. Therefore, assumption of field independent charge carrier mobility is dropped and *μ*(*E*) dependence is still valid in the absence of traps then it can be described by Poole-Frenkel(PF) equation^24^.

3 $$\mu (E)={\mu }_{0}\,\exp (\beta \sqrt{E})$$

This equation is more suitable to describe transport in organic materials^25,26^ which traps are often discovered in amorphous molecular materials, almost conjugated polymers and molecularly doped polymers^27^. In Eq. (3), *μ* depends on E, *μ*_0_ is the zero-field mobility and *β* is PF factor. The values for *μ*_0_ range from 10^−9^ to 10^−7^ *cm*^2^/*Vs*, while *β* is of the order of 10^−2^ (*cm*/*V*)^1/2^, under usual OLED fabricating conditions, the electron mobility is between 10^−6^ and 10^−5^ *cm*^2^/*Vs*^28^.

**Figure 5:**
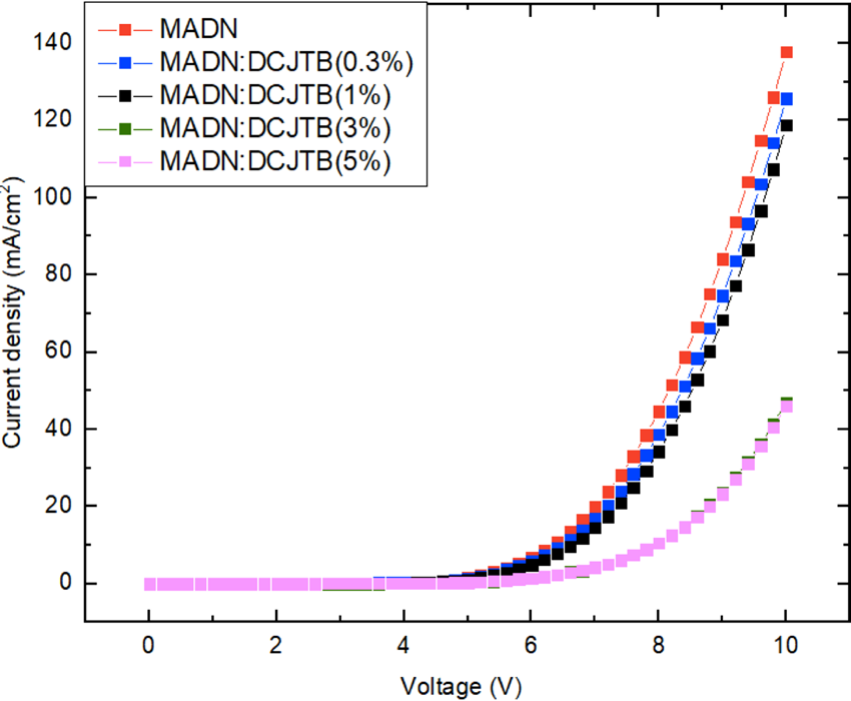
Characteristics curves of current density as a function of bias voltage for the devices at undoped MADN and various dopant concentrations.

Table 2 summarizes the carrier mobility of each organic material used in the devices and the carrier mobility value of NPB, MADN and TPBi were obtained from literatures^29–31^. As evident in Fig. 5, current density decrease with increasing the DCJTB dopant concentration. This result shows the charge transport in a host-dopant system is dependent on not only a host molecule but also a dopant molecule^12,32^. Therefore, we neglected the effect of dopant since the concentration rate of DCJTB is less than 1% weight in hosts. On the other hand, the doping concentration of BUBD-1 is 7% and it might affect the carrier mobility however, we did only use host mobility due to the lack of BUBD-1 doped carrier mobility data. Although with these difficulty, the empirical equation is still adequate and the aim of this study would be not affected^33,34^.

**Table 2:** The carrier mobility values of the organic materials used in devices^29–31^.

Materials	Carrier Type	Thickness(nm)	Mobility(*cm*^2^*V*^−1^*s*^−1^)^*^ at 5 V
NPB	Hole	80 nm	3.0 × 10^−5^
MADN	Hole	40 nm	1.7–2.0 × 10^−7^
MADN	Electron	40 nm	1.7 × 10^−7^
TPBi	Electron	5 to 50 nm	3.0–4.52 × 10^−5^

*Electric field = 500–600 (*V*/*cm*)^1/2^.

The thickness of TPBi(ETL) and NPB(HTL) thickness versus the BUBD-1 emission ratio indicates interference effects at different wavelengths which can be considered as a Fabry-Pérot cavity. From this phenomenon, it is possible to deduce the layer thickness through the following relation^35^.

4 $${d}_{1}=\frac{{\lambda }_{1}{\lambda }_{2}}{2n({\lambda }_{2}-{\lambda }_{1})}$$

where *λ*_1_ and *λ*_2_ are the shorter and longer wavelengths corresponding to the spectrum of each layer and *n* is refractive index of each layer (*n*_*TPBi*_ = 1.727, *n*_*NPB*_ = 1.733).

We can obtain an Eq. (5) simply etimated arrival time of holes and electrons from each electrode using a well know equation of v = d/t. We can deduce the carrier time of arrival (*t*) where *ν*_*d*_ is the carrier drift velocity derived from Eq. (2), *d*_2_ is the thickness of the organic layers and *d*_1_ is derived from Eq. (4).

5 $$t=\frac{{d}_{2}-{d}_{1}}{{v}_{d}}$$

*t* is the carrier arrival time which marks when carriers reach the recombination zone in EML whose physical location is reference to end of each HTL and ETL and use the carrier mobility of each layers was used to do the calculation. The Eq. (5) delineated approximate location of recombination zone since *ν*_*d*_ is fixed and the only variable is ETL thickness. These OLED devices were improved the roughness of the anodic electrode by plasma treatments but occurred interference effects in the device emission spectra. However, the micro-cavity is smaller than the thickness of the organic layers when the viewing angle is at 0° and it does not affect the CIE coordinates and EL of the device^36,37^.

**Figure 6:**
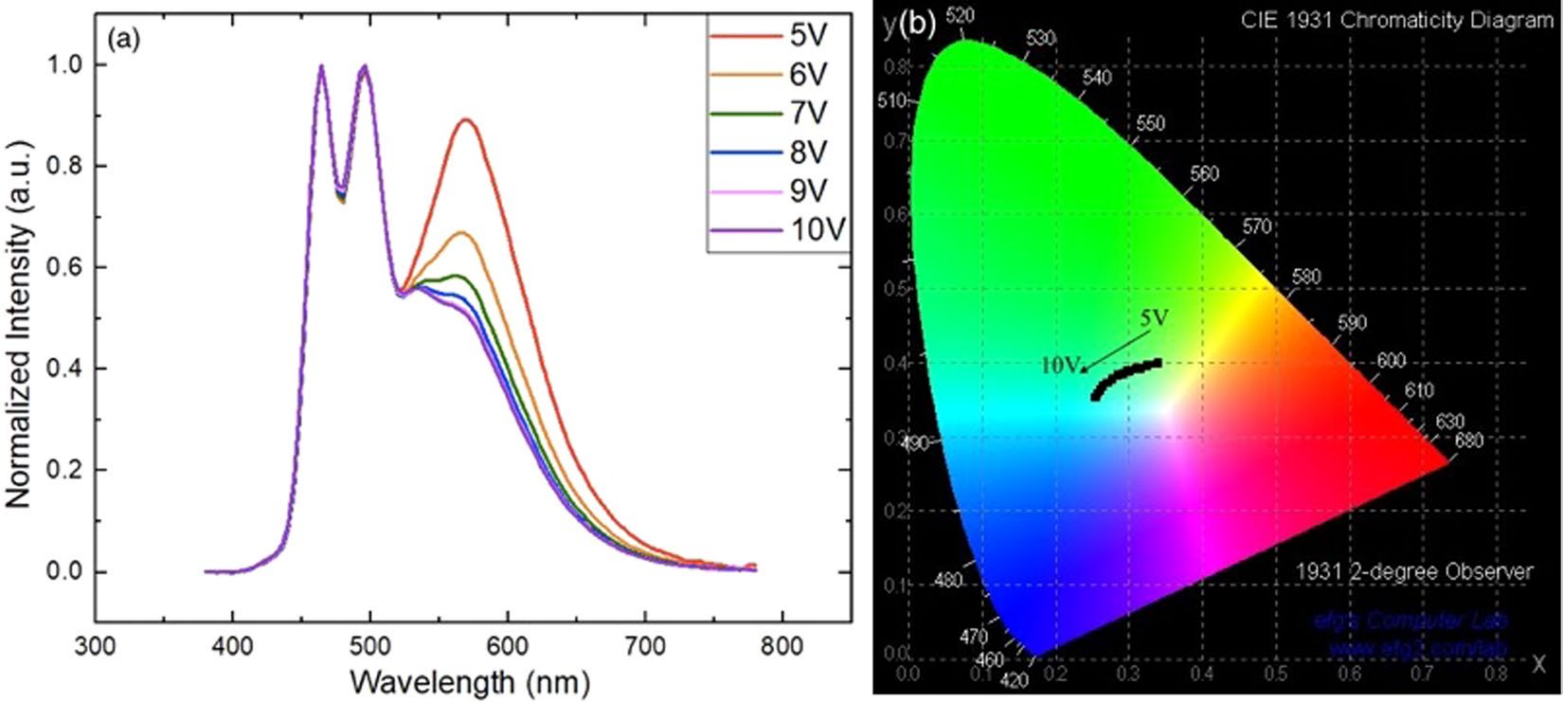
Voltage dependent spectra of (**a**) Device B1 measured at 5–10 V on a 1 V interval. (**b**) Color transitions of Device B1 plotted in CIE 1931 coordinates^38^.

There are also other conditions to various emitting ratio rates. At this time, we used fixed layer thickness and only depending on the driving voltage to observe the difference. As shown in Fig. 6, all spectra were normalized to the blue emission peak at 464 nm and 494 nm. When the driving voltage increased, the exciton recombination interface shifted closer to the blue EML, due to more electrons transported into the blue EML across the red EML, thus the red emission decreases with applied voltage and the CIE coordinates of Device B1 changes from warm white (0.34, 0.40) to cool white (0.27, 0.36). It also achieved high efficiency and stability in the Device B1. However, in this study, we focused on changing thickness of HTL and ETL to control the various emission ratio rate, so we could approximately calculate the recombination zone thus utilizing different driving voltage combined with this study could be a result of color tunable custom-built WOLEDs^39,40^.

**Figure 7:**
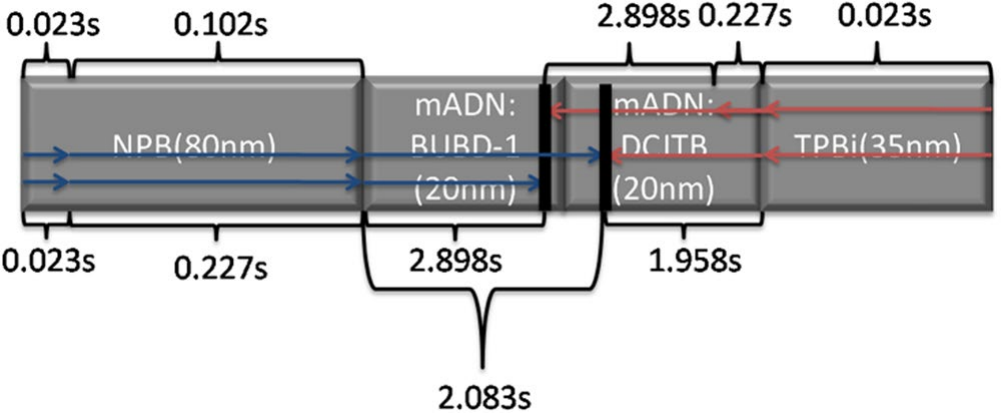
Schematic representation of carrier recombination location on emissive layers using the carrier arrival time Eq. (5) with Device C1 and this result is outlined in Table 3 (Blue line: holes, Red line: Electrons).

As shown in Fig. 7, we used Eq. (5) to obtain the carrier recombination time on the emissive layers and that indicate the actual location of recombination zone as shown in Table 3.

**Table 3:** The color coordinates of CIE with different emission ratios of BUBD-1(Blue) and DCJTB (Red) in WOLED and the location of recombination zone with different emission ratios of BUBD-1(Blue) and DCJTB (Red).

Emission Ratio(%) WOLED (BUBD-1:DCJTB)	Color coordinates of CIE of WOLED	Recombination zone (nm) (distance is with respect to the edge of BUBD-1 and DCJTB) BUBD-1/DCJTB
(Device A1) 78%: 22%	(0.21, 0.33)	17.43 nm/1.15 nm
(Device B1) 61%: 39%	(0.34, 0.40)	18.5 nm/1.32 nm
(Device C1) 46%: 54%	(0.46, 0.41)	19.27 nm/1.41 nm
(Device D1) 31%: 69%	(0.54, 0.42)	19.88 nm/1.54 nm

Normally, electron injection and transport layers have higher electric field than hole injection and transport layers, which mean electrons are transporting faster than holes. Figure 7 shows graphically where holes and electrons are arriving in recombination zone. Table 3 details the results of approximate carrier recombination zone with different emission ratios. To determine the location of recombination zone, we employed Eq. (5), the carrier recombination time (*t*) to select the recombination zone on each side of hole and electron transport layers including emissive layers. Throughout estimating carrier recombination time (*t*), we can define a location of the carrier recombination zone on emissive layer according to ETL thicknesses.

**Figure 8:**
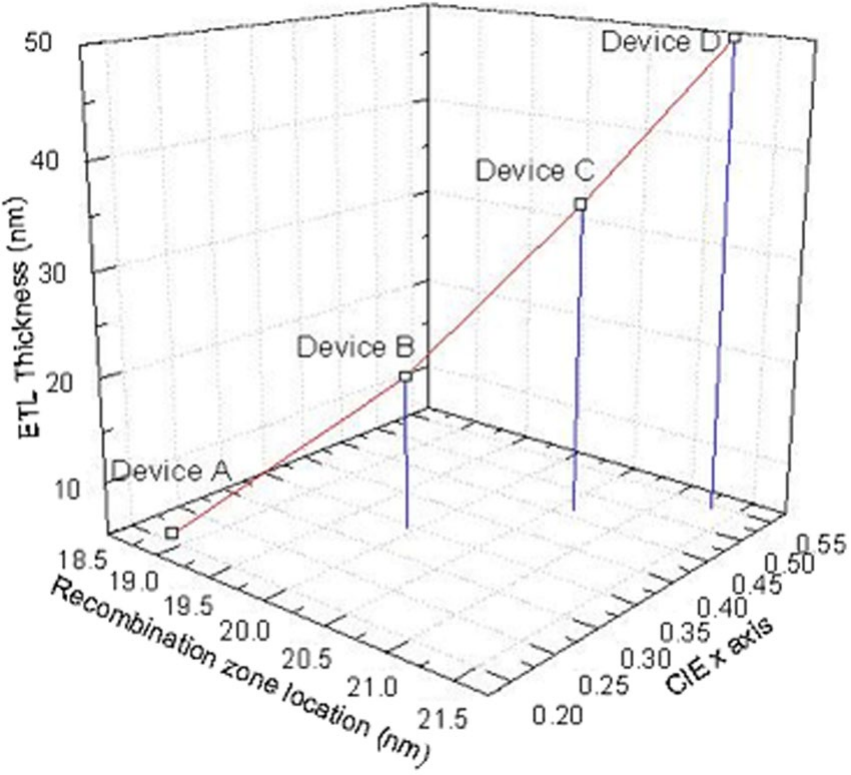
The plot of recombination zone(nm) vs CIE x axis by increasing the ETL(TPBi) thickness(nm).

As shown in Fig. 8, we have found the correlation between location of recombination zone, the CIE x axis and ETL thickness. It derived from Eq. (5) to calculate the recombination zone as shown in Fig. 7 and add up the recombination zone of the edge of BUBD-1 and DCJTB. With the added value, we derived an empirical equation through an exponential function graph substitution provided by scientific graphing and data analysis software: Origin based upon data from Table 3 and Fig. 8. The empirical equation is described as Eq. (6) in which *α* indicated the CIE x axis, A is the constant, x is the recombination zone location, and *d*_*etl*_ is the thickness of the ETL.

6 $$\alpha =A\ast \exp (-x/{d}_{etl})$$

From this result, we are able to design our WOLED structure in order to achieve desired emission ratio by approximating the location of the recombination zone. In other words, it allows us to custom-made WOLED in a more convenient way based upon device structure.

## Discussion

In this work, we obtained a preliminary relationship between thickness of electron/hole transport layer and ratio of two emission peaks. Also, the result shows the correlation between the emission ratio of BUBD-1/DCJTB and color coordinates of CIE in the emissive layer. The electron transport layer thickness is critical in the empirical equation:*α* = *A* * exp(−*x*/*d*_*etl*_).

From these results, the equation can be used to predict the emission ratio of WOLEDs which apply the desirable CIE x axis and recombination zone location to the equation and the thickness of ETL. Therefore, we can design the devices with different emission ratio using proper thickness of the organic layers. However, this equation can be used only the certain organic layer structure that used on this research due to various mobility on each organic materials. This quantitative analysis of charge distribution would allow us to fabricate custom-built WOLEDs in near future.
